# Complete Genome Sequence of Abscisic Acid-Metabolizing Rhizobacterium *Rhodococcus* sp. Strain P1Y

**DOI:** 10.1128/MRA.01591-18

**Published:** 2019-04-11

**Authors:** Natalia E. Gogoleva, Yevgeny A. Nikolaichik, Timur T. Ismailov, Yuri A. Khlopko, Svetlana A. Dmitrieva, Tatiana A. Konnova, Taras S. Ermekkaliev, Vera I. Safronova, Andrey A. Belimov, Yuri V. Gogolev

**Affiliations:** a Kazan Institute of Biochemistry and Biophysics, FRC Kazan Scientific Center of RAS, Kazan, Russian Federation; b Institute of Fundamental Medicine and Biology, Kazan (Volga Region) Federal University, Kazan, Russian Federation; c Department of Molecular Biology, Belarusian State University, Minsk, Belarus; d Institute for Cellular and Intracellular Symbiosis, Ural Branch of Russian Academy of Sciences, Orenburg, Russian Federation; e All-Russia Research Institute for Agricultural Microbiology (ARRIAM), Saint Petersburg, Russian Federation; University of Maryland School of Medicine

## Abstract

Mechanisms of microbial catabolism of phytohormone abscisic acid (ABA) are still unknown. Here, we report the complete genome sequence of ABA-utilizing Rhodococcus sp. strain P1Y, isolated from the rice (Oryza sativa L.) rhizosphere. The sequence was obtained using an approach combining Oxford Nanopore Technologies MinION and Illumina MiSeq sequence data.

## Abstract (precis)

Mechanisms of microbial catabolism of phytohormone abscisic acid (ABA) are still unknown. Here, we report the complete genome sequence of ABA-utilizing Rhodococcus sp.

## ANNOUNCEMENT

Representatives of the *Rhodococcus* genus can both produce and metabolize phytohormones and their precursors (1, 2). The strain *Rhodococcus* sp. P1Y was initially isolated from the rhizosphere of rice (Oryza sativa L.) seedlings using a selective nutrient medium containing abscisic acid (ABA) as the sole carbon source (3). Here, it was cultivated as described previously (3). Genomic DNA was extracted and purified using the QIAamp DNA minikit (Qiagen GmbH). Paired-end and mate pair libraries were prepared using the NEBNext Ultra II DNA library kit and the Nextera mate pair library prep kit (Illumina). Sequencing was performed on an Illumina MiSeq instrument with the MiSeq reagent kit v.2 (500 cycles), which generated 2,848,635 and 100,034 reads, respectively. The paired-end reads were filtered and trimmed with PrinSeq lite v.0.20.4 (4), leaving 2,704,317 high-quality read pairs (179× genome coverage). The mate pair reads were processed with NxTrim v.0.4.2 (5), leaving 65,022 proper mate pairs (6× genome coverage).

A Nanopore library was prepared using a 1D ligation sequencing kit (SQK-LSK-108; Nanopore) and sequenced on a MinION Mk1 device. The sequencing output was 4.3 Gb (49,975 reads with a mean length of 5.5 kb and maximal length of 175.1 kb; the predicted genome coverage depth was 64×). Nanopore data assembly was performed with Canu v.1.7, which produced a single circular contig (6). Single-nucleotide polymorphisms (SNPs), short indels, and local misassemblies remaining in the assembly were fixed by Pilon v.1.22 (7) using both paired-end and mate pair Illumina data mapped onto the assembled contig by BWA-MEM (8). Multiple rounds of error correction, using the default settings of Pilon and BWA, were performed until no more errors could be fixed. The absence of large misassemblies was confirmed with mate pair data and NxRepair (9).

The complete genome sequence of *Rhodococcus* sp. strain P1Y consists of a single circular chromosome of 5,868,661 bp with a GC content of 63.19%. Annotation was performed using the NCBI Prokaryotic Genome Annotation Pipeline v.4.5 (10). A total of 5,453 genes were identified, of which 5,253 were protein-coding genes, 138 were pseudogenes, and 62 were RNA genes, of which 12 were rRNA, 47 were tRNA, and 3 were noncoding RNA (ncRNA) genes.

The relationship of *Rhodococcus* sp. P1Y with other *Rhodococcus* spp. was assessed by calculating identities between their 16S rRNA genes. The sequences were compared to similar sequences in the NCBI database using BLAST analysis. The highest identity, at 98.62 to 98.75%, was with Rhodococcus fascians strain D188 (11). The average nucleotide identity between these genomes, calculated using JSpecies (12), was 74.4 to 74.7%.

### Data availability.

The complete genome sequence was deposited in GenBank under the accession number CP032762, corresponding to the sample accession number SAMN10180271. The raw read files were deposited in the SRA under the accession numbers SRX5005340, SRX5005339, and SRX5005338. The version described in this announcement is the first version, CP032762.1.
